# Self-reported effects of infertility on marital relationships among fertility clients at a public health facility in Accra, Ghana

**DOI:** 10.1186/s40738-015-0002-5

**Published:** 2015-07-01

**Authors:** Samuel H. Nyarko, Hubert Amu

**Affiliations:** 1 Department of Population and Behavioural Sciences, School of Public Health, University of Health and Allied Sciences, Hohoe, Ghana; 2 Department of Population and Health, University of Cape Coast, Cape Coast, Ghana

**Keywords:** Self-reported, Infertility, Marital relationship, Fertility clients

## Abstract

**Background:**

There is little empirical literature on the effects of infertility on marital relationships in Ghana. This study sought to examine the effects of infertility on marital relationship among fertility clients at a Public Hospital in Accra, Ghana.

**Results:**

The study revealed that infertility negatively affected the sexual life of participants as they generally reported that even though they still had regular sexual intercourse with their partners after realising they were infertile (61.6 %), sex was only for the purpose of reproduction and not for mutual sexual satisfaction (68.8 %). Sexual intercourse was reported to be unfulfilling as well as unenjoyable (64.3 %). The psychological well-being of participants (87.5 %) and stability within marital unions were also negatively affected by infertility, resulting in quarrels (72.3 %).

**Conclusions:**

Infertility has numerous negative implications for marital relationship. Thus, infertile persons should not be only physically examined and treated for infertility, but should also be given counselling to lessen the psychological trauma attached to infertility.

**Electronic supplementary material:**

The online version of this article (doi:10.1186/s40738-015-0002-5) contains supplementary material, which is available to authorized users.

## Background

Childbearing and rearing are important events in the life of every human and are positively associated with the ultimate goals of completeness, happiness and family integration [1]. Human existence reaches completeness through a child and fulfils an individual’s need for reproduction. Roupa et al. [1] describe infertility as the inability to procreate after trying for 1 year without the use of birth control methods while having normal sexual intercourse. The World Health Organisation [2] also describes infertility as the inability of a sexually active, non-contraception using couple to achieve pregnancy in 1 year.

Even though male infertility is acknowledged to exist, women are ultimately held responsible for a couple’s inability to reproduce [3]. Hence, a woman who has no child in the African setting for instance, is regarded as having no place in society. Infertility is a well-known public health issue in Africa. In Ghana, for instance, infertility is often attributed to witchcraft, physical abnormalities with spiritual origins, and sexual promiscuity [3–5].

According to Cooper [6], a couple’s sexual relationship is often the area of their life that is most negatively affected by infertility. Love making, which initially in marriage is a warm, loving, intimate and physically pleasurable experience becomes a dreaded chore, serving only as a means to an end and even continues to result in futility. To worsen situations, sex often becomes the battleground where a couple’s fears, anxieties and depressions are played out [6].

Psychological bonding is significantly related to quality of marital relationship [5]. However, when there is infertility in a marital union, the psychological bond between the couple, wanes away, resulting in negative psychological implications, including quarrels and fights in the union [6]. Infertility has been linked to conflicts in marital relationships [3]. Couples may avoid interactions with their friends particularly those who are pregnant and families who have children but can entirely not avoid conflict in the relationship arising due to their infertile status [4].

A considerable number of studies have been conducted on infertility in recent years. Padma [7] conducted a study in selected areas at Raichur where he investigated knowledge of infertile couples regarding infertility. Namujju [8] also examined knowledge, attitudes and practices towards infertility among adults 18–40 years in Kalisizo, in Uganda. The National Institute for Health and Clinical Excellence [9] also assessed the treatment of people with fertility problems, while Fledderjohann [3] examined the general implications of infertility in Ghana. Little is however known in Ghana in terms of existing literature on the effects of infertility on marital relationships. Consequently, this study sought to examine the effects of infertility on marital relationships among infertility clients at a Public Hospital in Accra, Ghana, in terms of sex life, psychological well-being, communication as well as conflicts and to determine whether any of the background characteristics is associated with psychological trauma due to infertility.

## Methods

Descriptive cross-sectional design was used for the study. All consenting heterosexual males and females who were married, and who sought care at the facility from September 1 to October 31, 2014 were included in the study. It however excluded clients who came to fertility clinic but were divorced as the study is focused on persons who were still in their marital relationships at the time of the study. The participants were interviewed individually, and not as couples. Statistics available at the health facility indicated that as at August 31, 2014, there were 123 fertility clients at the health facility. A census was conducted for all the clients as participants. However, only 112 clients finally participated in the study since 11 of them could not be reached or contacted for the study.

A self-developed questionnaire was used for collecting data for the study (see Additional file 1). The instrument showed high validity with a Cronbach’s alpha of 0.85. The questionnaire was divided into 5 sections: A to E. Section A comprised background characteristics of participants consisting of age, educational level, religion and ethnicity. Section B focused on self-reported effects of infertility on sex life. Issues considered were regularity of sexual intercourse, sex only becoming an act for procreation and not for mutual satisfaction, and the nature of sexual intercourse, after the realisation of infertility in the union. Section C dealt with self-reported effects of infertility on psychological well-being of participants. Psychological trauma due to infertility, expression of the psychological trauma and contemplation of suicide were the issues considered in Section C. Section D centred on infertility and communication while Section E focused on effects of infertility on the stability of marital relationships. This section considered quarrels, fights and threats of divorce in marital relationships due to infertility. The questionnaires were given to participants with formal education to fill by themselves, while the questions were read to participants without formal education to respond to them.

Ethical approval for the study was waived by the University of Cape Coast Ethical Review Board because it was an undergraduate study. Institutional approval was obtained from the hospital before data were collected from participants. Informed consent was sought from participants before including them in the study. This was achieved by explaining the purpose of the study to them and giving them informed consent forms to sign. Study participants were made aware that they had the right to discontinue with the process should they feel uncomfortable, and not to respond to questions that infringed upon their privacy.

Statistical Package for the Social Sciences (SPSS) version 21 was used to process the data. Results were presented in frequency tables and a diagram. Chi-square test was used to determine association between psychological trauma, contemplation of suicide and background characteristics of participants at 0.05 significance level.

## Results

### Background characteristics of participants

Background characteristics included in this study were age, sex, marital status, religion and ethnicity. From Table 1, the results of the study indicate that 73.2 % of the participants were aged 20 to 29, followed by those aged 30 to 39 with 20.5 %. Only a few (6.3 %) were aged 40 to 49. About 90 % of the participants were females and only about one-tenth (9.8 %) were males. Also, more than half (56.3 %) of the participants had secondary or higher education while about a quarter (25.9 %) had primary school education and only about 18 % had no formal education. With regard to religious affiliation, 87.5 % of the participants were Christians while 8.9 % were Muslims. Only 3.6 % of the participants were affiliated to African traditional religion. In terms of ethnicity, Ga-Adangmes were the dominant among the study participants with 39.3 %. This was followed by Ewes with 26.8 % with the Guans forming the least (2.7 %) of the participants of the study.

**Table 1:** Background characteristics of participants

Characteristic	Frequency	Percentage (%)
Age		
20–29	82	73.2
30–39	23	20.5
40–49	7	6.3
Sex		
Male	11	9.8
Female	101	90.2
Educational level		
No formal education	20	17.8
Primary education	29	25.9
Secondary education +	63	56.3
Religion		
Christian	98	87.5
Muslim	10	8.9
African traditionalist	4	3.6
Ethnicity		
Akan	24	21.4
Ga/Adangme	44	39.3
Ewe	30	26.8
Mole-Dagbani	11	9.8
Guan	3	2.7
*N*=	112	100.0

Source: Field work, 2014

### Self-reported effects of infertility on sex life in marital relationships

In order to ascertain the effects of infertility on sexual life in their relationships, participants were asked to indicate whether they had regular sexual intercourse, the main purpose of sexual intercourse and to describe the nature of sexual intercourse in their marital relationships after realising they were infertile (Table 2). With regard to regularity of sexual intercourse, about 62 % of the participants reported that they had always had regular sexual intercourse with their partners even after the realisation of infertility while about 38 % reported otherwise. In terms of the major purpose of sexual intercourse, about 69 % of the participants reported that sexual intercourse was mainly for procreation but not for mutual satisfaction while about 31 % reported that sex was not only for procreation but also for the mutual satisfaction of both partners. Regarding the nature of sexual intercourse, about 64 % reported that sexual intercourse was unfulfilling and unenjoyable while about 34 % reported that it was fulfilling and enjoyable with only about 2 % reporting that they did not know.

**Table 2:** Self-reported effects of infertility on sex life in marital relationships

Statement	Frequency	Percentage (%)
Regular sexual intercourse		
Yes	69	61.6
No	43	38.4
Sex is only for procreation but not for mutual satisfaction		
Yes	77	68.8
No	35	31.2
Nature of sexual intercourse		
Unfulfilling and unenjoyable	72	64.3
Fulfilling and enjoyable	38	33.9
Don’t know	2	1.8

Source: Field work, 2014

### Self-reported effects of infertility on the psychological well-being of individuals

As indicated in Table 3, 87.5 % of the participants reported that they experienced psychological trauma due to infertility. Among those who had experienced psychological trauma, about 41 % reported that they expressed their trauma through crying for days without eating while about 45 % reported that they usually blamed themselves for being infertile and only about 14 % reported picking up quarrels with people over the least provocation. Also, 88.4 % of the participants reported that they did not contemplate suicide due to their infertility while only 11.6 % reported that they had contemplated suicide.

**Table 3:** Self-reported effects of infertility on psychological well-being of individuals

Statement	Frequency	Percentage (%)
Psychological trauma due to infertility		
Yes	98	87.5
No	14	12.5
Expression of psychological trauma (*n* = 98)		
Crying for days without eating	40	40.8
Blaming one-self for being infertile	44	44.9
Quarrelling with people over the least provocation	14	14.3
Contemplation of suicide		
Yes	13	11.6
No	99	88.4

Source: Field work, 2014

The study therefore examined the association between psychological trauma and background characteristics of participants using chi-square test (Table 4). As indicated in Table 4, psychological trauma due to infertility had no association with age (*p* = 0.64), sex (*p* = 0.12), educational level (*p* = 0.36), religious affiliation (*p* = 0.11) and ethnicity (*p* = 0.90) at 0.05 significance level. The study further examined the association between contemplation of suicide due to infertility and background characteristics of participants (Table 5). From Table 5, it can be observed that there was no association between contemplation of suicide and participants’ age (*p* = 0.49), sex (*p* = 0.15), religious affiliation (*p* = 0.115) as well as ethnicity (*p* = 0.51) at 0.05 significance level. Only level of education (*p* = 0.00) had an association with participants’ contemplation of suicide due to infertility.

**Table 4:** Chi-square test of association between psychological trauma and background characteristics of participants

Background characteristics	Chi-square (*X* ^2^)	*P*-value
Age	0.899	0.64
Sex	2.378	0.12
Educational level	0.855	0.36
Religious affiliation	4.475	0.11
Ethnicity	1.058	0.90

Source: Field work, 2014

Significance level ≤0.05

**Table 5:** Chi-square test of association between contemplation of suicide and background characteristics of participants

Background characteristics	Chi-square (*X* ^2^)	*P*-value
Age	1.443	0.49
Sex	2.081	0.15
Level of education	9.392	0.00*
Religious affiliation	4.323	0.12
Ethnicity	3.296	0.51

Source: Field work, 2014

*Significant at 0.05 significance level

### Self-reported effects of infertility on communication in marital relationships

**Fig. 1:**
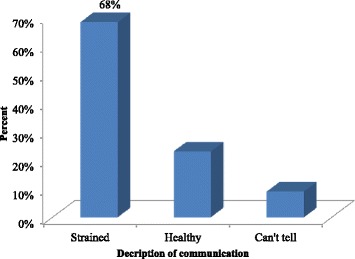
Nature of communication in marital relationship. Source: Field work, 2014

From Fig. 1, majority of the participants (68 %) described communication as strained while 23 % described it as healthy. Participants constituting 9 % could however not tell whether communication in their relationships was strained or healthy.

### Effects on stability of marital relationships

The study further sought to assess the effects of infertility on the peace and stability in marital relationships of participants. This was assessed in terms of existence of quarrels, fights and divorce threats using a 3-point Likert scale statements. A summary of the results is presented in Table 6. From the table, about 72 % of the participants agreed that they had quarrelled with their partners over their infertility. About 18 % however disagreed that they had quarrelled with their partners due to their infertility. Only a few (9.8 %) were quite uncertain with the statement. Also, more than half (54.4 %) of the participants disagreed that they had fought with their partners due to infertility while 42 % however agreed to the statement and 3.6 % were uncertain. In a similar way, more than half (55.4 %) of the participants disagreed that their partners had threatened to divorce them due to their infertility. About 40 % however agreed that their partners had threatened them with divorce while only a few were not certain.

**Table 6:** Effects on stability of marital relationships

	*N* = 112	Participants’ agreement (%)	
Statement	Agree	Uncertain	Disagree
You have ever quarrelled with your partner over your infertility	72.3	9.8	17.9
You have ever fought with your partner over your infertility	42.0	3.6	54.4
Your partner has ever threatened to divorce you due to your infertility	40.2	4.5	55.4

Source: Field work, 2014

## Discussion

The study found that more females than males, attended infertility clinic at the health facility. This echoes Fledderjohann’s [3] argument that females are usually blamed for infertility. As such, they were mostly the persons who sought treatment for the couple’s inability to reproduce. The study found that even though the participants had regular sexual intercourse, sexual intercourse between the couples was merely for procreation purposes but not for mutual satisfaction. The majority of the participants reported that sexual intercourse was unfulfilling and not enjoyable. This finding is consistent with findings of Zegers-Hochschild et al. [10] that when there is infertility in a marital relationship, love making which initially is an intimate, warm, loving and pleasurable act can then become just a means to an end, since the only focus becomes reproduction, which even fails. Thus, love making among couples became devoid of care, intimacy, emotional attachment and reciprocity that may be required in achieving total mutual sexual satisfaction.

The study further found that the majority of the participants suffered psychological trauma due to their infertility. This was usually expressed through consistent cries and self-accusation or self-condemnation as well as being overly sensitive. Hence, infertility negatively affected the psychological well-being of participants. This echoes findings of a study conducted by Peterson et al. [11] in which the authors argued that infertility is negatively related to the psychological functioning of both women and men. It came out that even though majority of the participants experienced psychological trauma due to their condition, only a few contemplated or considered committing suicide as a permanent solution to the condition. Thus, most of the participants never contemplated suicide and this may be due to a number of factors including knowledge of treatment for the condition, education as well as possible availability of friends and relatives who may serve as source of comfort and encouragement for the participants.

The study therefore found no association between psychological trauma and any of the background characteristics of participants. This may imply that irrespective of the background of the participant, he or she may be well susceptible to psychological trauma as a result of infertility. It also came out that contemplation of suicide due to infertility was associated with only level of education of participants. This may be due to the fact that education may be a source of enlightenment concerning the various options of treatment for the condition. The study also revealed that communication in the marital relationships of participants was damaged. Hence, the assumption that communication between couples which has positive impacts on their marital relationships becomes questionable in the face of infertility where communication between the couple becomes strained [12].

Infertility was also found to have impinged on the stability of marital relationships by causing conflicts in the marital relationships of participants. Participants had misunderstandings with their partners over their inability to give birth to children even though majority had never fought with their partners or threaten them with divorce. This confirms the assertion made by Koenig et al. [13] that infertility has a link with conflicts in marital relationships. Tufts et al. [14] also note that marital relationships may suffer, especially, when couples are dealing with infertility and may thus avoid interactions with their friends particularly those who are pregnant as well as families who have children. Thus, couples cannot entirely avoid conflict in their marital relationships due to their infertile status [14].

## Conclusions

Infertility had a negative effect on the marital relationships of the infertile persons who attended the public health facility. Also, the sexual life, communication in the marital relationships as well as the psychological well-being of the participants was strained as a consequence of infertility. Conflicts resulting from infertility also derailed the stability of marital relationships of participants of the study. The temptation to commit suicide because of infertility was significantly associated with level of education of participants. The implication of this is that fertility clients do not only need treatment for their condition but also need to be given guidance and counselling to lessen the psychological trauma that most of them face, to improve communication and stability of their marital relationships as well as the sex life of infertile couples. The findings also underscore the importance of pre-nuptial fertility tests and other necessary tests in order to ascertain the fecundity as well as compatibility of potential couples before marriage.

## Additional file

Additional file 1:
**Questionnaire for clients with infertility.**
