# Supplementary material for: Self-reported effects of infertility on marital relationships among fertility clients at a public health facility in Accra, Ghana
Source: Fertil Res Pract. 2015 Jul 1;1:10. doi: 10.1186/s40738-015-0002-5 (PMC5424354; doi:10.1186/s40738-015-0002-5)
Supplement: Additional file 1: — Questionnaire for clients with infertility. [file 40738_2015_2_MOESM1_ESM.doc]

**QUESTIONNAIRE FOR CLIENTS WITH INFERTILITY**

This study is being conducted on **Self-Reported Effects of Infertility on Marital Relationships at the La General Hospital, Accra.** The researchers will be most grateful if you could participate in the survey as candidly as you can. You may decide not to respond to any question which makes you feel uncomfortable. You may also withdraw from the process if you so wish. No name is needed and your responses will be treated with utmost confidentiality.

Indicate your signature and date, if you agree to be part of this study.

____________________ _________________________

Signature Date

Thank you for accepting to be part of this Study

**Section A: Socio-demographic characteristics**

Tick [√] the appropriate boxes

1. Age (in completed years)
2. Below 20 [ ]
3. 20-29 [ ]
4. 30-39 [ ]

D. 40-49 [ ]

E. 50 and above [ ]

1. Sex
2. Male [ ]
3. Female [ ]
4. Level of education
5. None [ ]
6. Primary [ ]
7. Secondary and above [ ]
8. Religion
9. Christian [ ]
10. Muslim [ ]
11. African traditionalist [ ]
12. Other (specify) …………………
13. Ethnicity
14. Akan [ ]
15. Ga/Adangme [ ]

C. Ewe [ ]

D. Mole-Dagbani [ ]

E. Guan [ ]

F. Other (specify) ………………

1. How long have you been in your current marital union?

A. 0-12 months [ ] B. 1-2years [ ] C. 3-4years [ ] D. 5-6 years [ ]

E. 7-8 years [ ] F. 9-10 years [ ] G. More than 10 years [ ]

1. Have you ever been in another marital union prior to this one in which you brought forth children?
2. Yes [ ]
3. No [ ]

9. If yes to question 8, how many children did you have in that marital union?

A. None [ ] B. 1 [ ] C. 2 [ ]

D. 3 [ ] E. 4 [ ] F. More than four [ ]

**Section B: Self-reported Effects of Infertility on Sex Life in Marital Relationships**

1. How long has it been, since you and your partner realised that you were not giving birth to children and therefore decided to seek treatment?

A. 1-2years [ ] B. 3-4years [ ] C. 5-6 years [ ]

D. 7-8 years [ ] E. 9-10 years [ ] F. More than 10 years [ ]

1. Since you started seeking help, have you been regularly having sexual intercourse with your partner?

A. Yes [ ] B. No [ ]

1. How will you describe sexual intercourse with your partner since you realised you were not giving birth?
2. Unfulfilling and unenjoyable [ ]
3. Fulfilling and enjoyable [ ]
4. Don’t know [ ]
5. Has sex in your marital relationship become only an act for procreation but not necessary about providing the sexual needs of each other in your marital union?

A. Yes [ ] B. No [ ]

**Section C: Self- Report Effects of infertility on psychological functioning of individuals**

1. You have ever been blamed for your fertility problem?

A. Yes [ ] B. No [ ]

1. If you have ever been blamed for your fertility problem, which of the following people were responsible for blaming you? (Tick as many as applicable)

A. Friends [ ] B. In-laws [ ] C. Spouse [ ] D. Neighbours [ ]

E. Other (specify) …………………………………..

1. Do you normally feel psychologically traumatised because of your fertility problem?

A. Yes [ ]

B. No [ ]

1. If your answer to question 16 is yes, how do you express your emotions?

A. Crying for days without eating [ ]

B. Blaming myself for being infertile [ ]

C. Quarrelling with people over the least provocation [ ]

C. Other (specify) …………………………………………………

1. Have you ever contemplated suicide as a result of your fertility problem?

A. Yes [ ]

B. No [ ]

**Section D: Self-reported effects of infertility on communication in marital relationships**

1. Your level of communication with your partner has reduced after you realised you were not giving birth.

A. Strongly Agree [ ] B. Agree [ ]

C. Disagree [ ] D. Strongly Disagree [ ]

1. You still discuss your work (if any) with your partner, and he or she is usually receptive with such discussions?

A. Strongly Agree [ ] B. Agree [ ]

C. Disagree [ ] D. Strongly Disagree [ ]

1. How will you generally describe communication in your marriage currently?

A. Healthy [ ]

B. Strained [ ]

**Section E: Self-Reported effects of Infertility on Stability of Marital Relationships**

1. You have ever quarrelled with your partner over your infertility.

A. Agree [ ]

B. Uncertain [ ]

C. Disagree [ ]

1. You have ever fought with your partner over your infertility.

A. Agree [ ]

B. Uncertain [ ]

C. Disagree [ ]

1. Your partner has ever threatened to divorce you due to your infertility.

A. Agree [ ]

B. Uncertain [ ]

C. Disagree [ ]

1. There has ever been a misunderstanding in your home which you can easily attribute to your infertility.
2. Agree [ ]
3. B. Uncertain [ ]
4. C. Disagree [ ]
